# Study of the principles in the first phase of experimental pharmacology: the basic step with assumption hypothesis

**DOI:** 10.1186/s40360-019-0306-x

**Published:** 2019-05-21

**Authors:** Yilkal Tariku Belay

**Affiliations:** School of Biomedical Sciences, College of Health Sciences, Makerere University, Kampala, Uganda

**Keywords:** Acute toxicology, Immunoassay, Dichlorvos, Chlorpyrifos, Cypermethrin

## Abstract

**Background:**

Experimental pharmacology deals with effects of various test substances studied on different animal species which is aimed at finding out safe therapeutic agent suitable for public health as well as mechanism and site of action of a test substance. It is the basic step in the discovery of new drugs or studying the pharmacological actions of already developed one using both preclinical and clinical study designs in a stepwise phase of investigations. However, the investigations in the first phase of experimental pharmacology are usually concluded with assumption hypothesis without any adequate validation of the scientific evidence. Single dose acute toxicology had been conducted on Balb c mice with three different level of doses prepared from each of three different test chemicals (Dichlorvos, Chlorpyrifos and Cypermethrin) with known median lethal dose (LD_50_) to define the fundamental principles, cause of toxicity and investigation timeframe in the first phase of experimental pharmacology.

**Methods:**

The methods used for data collection were: procurement of test chemicals, investigation of single dose acute toxicity on Balb c mice and quantitative immunoglobulins test. Data was thematically compiled for validation of the findings from each of the sources.

**Results:**

The result showed that the dose had never limited the toxic property of tested chemicals but the magnitude of adverse effect and length of time at which adverse effect was manifested on treated Balb c mice. The toxicity of tested chemicals was however limited by the toxic reaction rate of a dose in the biological process of exposed Balb c mice. The toxic effect of tested chemicals became magnified within a short period of time when large amount administered orally. It also remained after a long period of time when small amount administered in the same route.

**Conclusion:**

Adequate investigation time for acute toxicity study was therefore essential for comprehensive analysis of pharmacological property of tested chemicals at different level of doses.

## Background

Experimental pharmacology is a study through experimental design in controlled situations which involves testing of pharmacologically unknown substance and pharmaceutical products in human and animal [1]. It deals with effects of various test substances studied on different animal species which is aimed at finding out safe therapeutic agent suitable for public health as well as mechanism and site of action of a test substance [1].

Experimental pharmacology is the basic step in the discovery of new drug or studying the pharmacological actions of already developed one using both preclinical and clinical study designs in a stepwise phase of investigations [1]. It is a must to go through a number of critical steps in drug discovery and development effort to arrive at a compound that is safe and efficacious that also exhibits the desired drug quality or behaviour which warrants advancement to the clinic [1]. However, the investigations in the first phase of experimental pharmacology are usually concluded with assumption hypothesis without any adequate validation of the scientific evidence. It is mostly conducted in a biomedical laboratory setting where In vitro and In vivo study designs could be performed. An In vitro experimental study refers to a test which is taking place in a test tube, culture dish or elsewhere outside the living organism to evaluate the biological property of test material [1]. An In vivo experimental study is the opposite of In vitro which refers to an experimental study carried out within the living organism to investigate the pharmacological property of test material [1]. In vivo tests are usually conducted prior to In vitro tests to determine the toxicity of test material in which both studies are important steps in drug discovery.

Different species of laboratory animals are used in experimental pharmacology to investigate dose –biological response relationship and pharmacokinetic of different test substances. The laboratory animals mostly used are, Mice, Rat, Guinea pig and Rabbits [2]. Experimental study on Balb c mice had been conducted in the biomedical laboratory, department of pharmacology and therapeutics at Makerere University to answer the following questions. These are: (1) Does the dose determine toxicity of a chemical substance? (2) What make the toxicity of a dose? (3) Why different chemical substances with the same dose have different length of time at which its pharmacological effect manifested in treated study animal? These questions were once again answered through experimental investigation of test chemicals with known toxicity which is explained in detail in the result and discussion section of this study.

### Single dose toxicity study

Acute toxicity is the adverse effect produced after administration of a single dose of test substance using one of the routes of drug administration within a period of not exceeding 24 h [3]. It is usually conducted to support the development of new drug or medicine where the death of study subject is an end point. But, the use of lethal effect as an end point for conclusion makes acute toxicity study less valuable in safety regulatory measures. There is no a specific minimum lethal dose and maximum non-lethal dose for every test substance that can be manifested within 24 h. Different dose has different length of time at which it can cause significant pharmacological effect in treated subject [4]. The range of doses that can cause lethal effect to treated study animal also varies extensively because of strain, age and sex of the study animal and route of drug administration [5]. According to the current existing guidelines, however, the objective of acute toxicity study is to identify the dose which causes major adverse effects and an estimation of a minimum lethal dose within 24 h which has no adequate scientific grounds. It has limited value in terms of preclinical and human safety assessment due to the fact that the adverse effect of considerable test materials manifested after 24 and 48 h being administered to study subject [4].

Acute toxicity study is routinely conducted in experimental pharmacology as a regulatory requirement in order to avoid infiltration of harmful pharmaceutical products for public consumption [6]. But the routine investigation requirement for acute toxicity studies are not standing alone for validating the data because of assumption technical backgrounds [7]. There is no clinical pathology, immunology or other clinical measures conducted in acute toxicity studies to validate the data with adequate scientific background [8]. Acute toxicity study has still a controversy on both ethical and scientific grounds in which more animals are used in assessment of lethal end point study with limited scientific toxicity analyses. Death usually happens as a result of loss of bodyweight (wasting syndrome) from test substance induced inhibition of gluconeogenesis and appetite suppression [7].

If the objective of acute toxicity study in animal is to provide the primary safety data to aid in the selection of a compound for clinical development, the toxicity study should be well designed to assess dose- biological response relationship and pharmacokinetics in the treated subject through adequate length of investigation time. Clinical and histopathology need to be evaluated at the earlier and termination time of acute toxicology for adequacy of public health safety [9].

## Methods

### Research design

The study was an experimental study which was conducted on Balb c mice to analyse the research questions mentioned earlier in the background section. It has been analysed using one independent and two dependent research variables stated as follows respectively:

- Administered dose (*d*)
- Elapsed time (*t*) for adverse effect manifestation and
- The immune response (*ΔIg*)

An integrated biological approach (physiological and immunological analyses) was employed in this study to evaluate the different biological responses to test chemicals administered to Balb c mice. First, acute toxicity study was conducted in the biomedical laboratory of pharmacology and therapeutics during which the time elapsed for the manifestation of significant adverse effect on treated Balb c mice was determined and recorded in note book. The immune response had also been evaluated using quantitative immunoassay before dosing as reference test and at four hour after dosing for comparison from which changes in concentration of serum immunoglobulins*(ΔIg)* had been calculated and documented. The biological responses as toxic severity and toxic reaction rate of each doses administered to lab Balb c mice were finally determined using mathematical formula $\left(s=\frac{r}{d}\times 100\right)$ %/sec and $\left(r=\frac{d}{t}-\varDelta Ig\right)$ mg/sec respectively which was hypothesized in the previous study [13] where *s* is toxic severity, *r* is toxic reaction rate, *d* is the administered dose, *t* is the length of time at which adverse effect manifested and *ΔIg* is change in concentration of serum immunoglobulins at four hour after dosing. The computed result of toxic severity and toxic reaction rate were used to answer the research questions mentioned earlier which may help to define the fundamental principles in experimental pharmacology.

### Data collection methods

Three data collection methods were used. These were, procurement of test chemicals, investigation of single dose acute toxicity on laboratory Balb c mice and quantitative immunoglobulins test.

#### Procurement of test chemicals

Three toxic chemicals (Dichlorvos, Chlorpyrifos and Cypermethrin) with different LD_50_ [10, 11] were procured from agricultural chemical stores in Kampala, Uganda_._ Three different doses from each test chemicals (10, 50 and 90 mg/kg body weight) were prepared using micropipette and stored in separate and sterile, disposable test tubes which were pre-labeled with number and concentration of test chemical. The prepared doses have been stored at room temperature until it was tested on laboratory Balb c mice.

#### Procedures for acute toxicology

Data collection in acute toxicology has been conducted in the biomedical laboratory of department of pharmacology and therapeutics from 14th to 28th June 2018. First, fifteen female Balb c mice at the age of 10 weeks were procured from college of veterinary medicine, Makerere University and transported to the department of pharmacology and therapeutics where they have been kept in a cage by providing them food (bread and layer’s mash) and water in a daily basis. Even though, the toxicity of prepared test chemicals has been planned to be evaluated on 15 Balb-c mice, six mice were attacked and killed by a rat which was escaped from its cage at night during the experiment. As a result the experiment was evaluated on nine Balb c mice by dividing them in three groups by which three mice were selected by non-probability sampling, labeled at the head, back and thigh with different colours i.e. black, blue and red and kept them in separate cages. After acclimatising them in cages under standard environmental conditions of light and dark cycles for two weeks, about 1 ml of blood specimen for reference quantitative immunoglobulins test was drawn from the tail and facial veins of each Balb c mice using micro test tubes pre-labeled with number corresponding with the label of Balb c mice. After blood specimens drawn for reference quantitative immunoassay, three days have been given to all lab Balb c mice to restore normal bio-physiological state. And then, all sampled mice have been starved for overnight before weighing them and calculating the right dose. The mice from each group weighed separately using an electronic balance. First, the mouse picked from the cage by holding its tail and put in a plastic bottle with its tail off which was already measured. The weight of each mouse has been recorded in note book from which the right dose per body weight was calculated**.**

The calculated doses of prepared test chemicals were also measured using micropipette**.** Three different level of doses which were less than the LD_50_ [10, 11] (10, 50 and 90 mg/kg body weight) were prepared from each test chemicals and administered orally to the first, second and third group of 3 laboratory mice each using intragastral tube. The Dichlorvos, Chlorpyrifos and Cypermethrin have been tested on blue, black and red labeled mice respectively. The effect of each administered doses to Balb-c mice has been monitored 3 times a day (in the morning, at noon and in the evening) for 5 days by providing food (bread and layer’s mash) and water in daily basis. The adverse effect and time of onset after dosing have been recorded against the dose in note book.

#### Procedures for quantitative immunoassay

Quantitative immunoglobulins test was conducted on blood specimens drawn from the tail and facial veins of laboratory Balb c mice before treatment as reference test and four hours after treatment for comparison. First, the mouse has been picked by holding its tail and placed on the working surface and cup the free hand over the mouse, scruff it firmly using the thumb and first finger. The hairless freckle on the side of the jaw and belly of the tail was located where the facial and tail veins could be accessed respectively and pricked with the lancet. After quickly drop the lancet into the sharps container, about 1 ml of blood was collected using micro test tubes which was pre-labelled with number corresponding to the label of laboratory Balb c mice from which blood sample was drawn. The mouse released into the cage and the blood specimens were transported to the department of Biomolecular and Biosecurity, college of Veterinary Medicine, Makerere University to process the specimens for quantitative immunoglobulins test from 1st to 10th July 2018.

The blood specimens were centrifuged using micro centrifuge at ten thousand revolution per second for five minutes. The serum from each blood specimens was extracted and stored in a separate Eppendorf tube pre-labeled with number corresponding with the label of blood specimen. The serum in each Eppendorf tube has been kept in a refrigerator at 4^o^c until it was processed and sent to South Africa through Lancet Laboratories for immunoglobulin quantification tests. Each serum specimens was labeled with the age of the mouse from which blood sample was drawn and identification number, packed in a white polyethylene bag in which the specimens were transported to Lancet Laboratories through which all the specimens were again transported under ambient temperature to the mother laboratory in South Africa – LANCET SA by DHL. Tests were conducted using architect system – Abbot and results with lab reference number were issued to the principal investigator through e-mail.

### Data processing and analysis

Data from different data collection methods was recorded manually in notebooks, smartphone camera and computer soft copy. Then, the data was systematically arranged, processed and analysed manually and using a computer package (Microsoft office excel 2013, Microsoft office word 2013 and smartphone with calculator) for content. Subject matters of the study were identified and organized into meaningful categories and sub–categories. For comprehensiveness, data from different data collection methods was compiled to validate and complement the findings from each of the sources.

### Data presentation

The analysed data from each of the sources presented in form of tables, graphs and use of descriptive statements under the themes mentioned in the result and discussion sections.

### Ethical consideration

The researcher was given an introduction letter from the department of pharmacology and therapeutics, and ethical clearance from Internal Review Board of School of Biomedical Sciences, Makerere University to seek permission and conduct research. Heads of departments, where different sections of the study has been conducted, were contacted. The norms and rules of each department has been highly observed. Laboratory Balb c mice have been treated according to the universal declaration on animal welfare (UDAW) to prevent cruelty and reduce suffering [12].

## Results

### Report of single dose acute toxicology

**Fig. 1:**
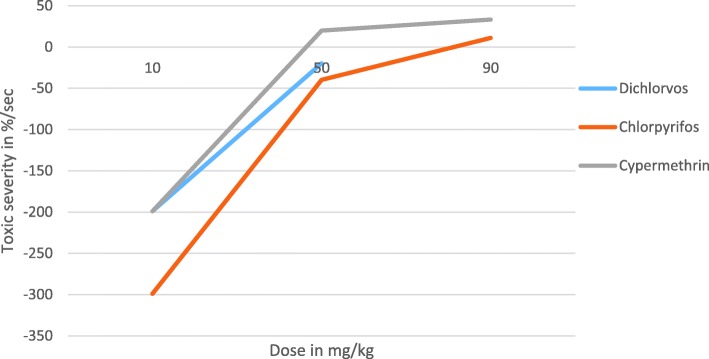
Toxic severity of tested doses prepared from Dichlorvos, Chlorpyrifos and Cypermethrin

**Fig. 2:**
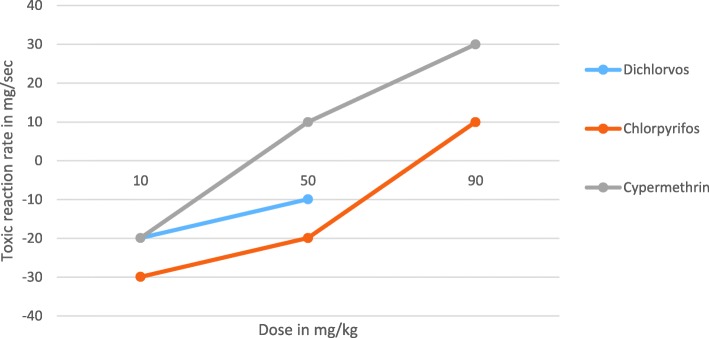
Toxic reaction rate of tested doses prepared from Dichlorvos, Chlorpyrifos and Cypermethrin

Doses of test chemicals prepared from Dichlorvos, Chlorpyrifos and Cypermethrin pesticides at three different level of doses (10, 50 and 90 mg/kg) had been administered into laboratory Balb c mice at 10 weeks old and monitored for a maximum period of 5 days. The length of time at which undesired biological effect manifested in treated Balb c mice was inversely related to the amount of dose administered in the oral route. The higher the dose of administered test chemical, the shorter the length of time at which adverse effect manifested on treated Balb c mice (Table 1). The previous study had also revealed that the dose had never limited the toxic effect of ethanol and ether test extracts from the dried seed of *Aristolochia elegns mast* but the length of time at which undesired effect was clearly manifested on treated Balb c mice [4]. The toxic effect of test extracts became magnified within a short period of time when large amount administered orally [4]. It also remained after a long period of time when small amount administered in the same route [4]. As a result, it was difficult to determine the lethal dose (LD_50_) and effective dose (ED_50_) of test extracts precisely [4]. This implies that the adverse effect of tested chemicals was not because of the dose but rather due to its toxic reaction rate which ultimately determined the toxic severity of tested chemical in the biological process of treated Balb c mice. The toxic reaction rate (*r*) and toxic severity (s) of each doses administered into Balb c mice in the oral route were calculated using mathematical formulation $\left(r=\frac{d}{t}-\varDelta Ig\right)$ mg/sec and $\left(s=\frac{r}{d}\times 100\right)$ %/sec respectively which are explained in detail in the discussion section and recorded in (Tables 2 and 3) [13]. The toxic severity and toxic reaction rate of Cypermethrin were more severe than Dichlorvos and Chlorpyrifos in the three level of doses prepared from each test chemicals and administered to lab Balb c mice in the oral route (Figs. 1 and 2). The toxic severity and toxic reaction rate of the two different level of doses prepared from Dichlorvos were also slightly more severe than Chlorpyrifos. However, the toxic severity of each test chemicals was not linearly projected as the dose administered to lab Balb c mice uniformly increased due to differences in the strength of the immune response (Fig. 1). The toxic severity of doses at 50 and 90 mg/kg prepared from Cypermethrin for instance, had disproportioned difference which was administered to different Balb c mice that had different strength of immune response. The toxic reaction rate of doses at 10 and 50 mg/kg prepared from Dichlorvos and Chlorpyrifos was slightly different which was not proportional to the dose administered to Balb c mice in the oral route (Table 3). The study revealed that the higher the strength of the immune response, the less toxic severity of test chemical in the biology of treated Balb c mice (Table 4 and Fig. 1). The strength of immune response of Balb c mice treated with the dose at10 mg/kg of Dichlorvos was greater than the Balb c mice treated with the dose at 50 mg/kg of the same test chemical which caused much less toxic severity to treated Balb c mice. This means that degree of toxic severity of tested chemicals was affected not only by the dose and its toxic reaction rate but also by the strength of immune response of Balb c mice. The degree of toxic severity was more affected than toxic reaction rate by the strength of the immune response of treated Balb c mice (Figs. 1, 2 and Tables 2, 3, 4).

**Table 1:** The length of time at which adverse effect significantly manifested on Balb c mice treated with test chemicals orally

Test chemical	Doses tested	№ of Mice	Weight in *gm*	Time at which test substance administered	Time at which signs of adverse effect clearly manifested	Duration
1.1.Dichlorvos	10 mg/kg	1	15.13	10:22	11:22	1 h
	50 mg/kg	1	17.63	10:23	10:53	30 min
	90 mg/kg	1	16.42	10:24	10:39	15 min
1.1.Chlorpyrifos	10 mg/kg	1	30.41	10:28	13:00	2:30 h
	50 mg/kg	1	27.12	10:29	12:00	1:30 h
	90 mg/kg	1	26.84	10:30	11:00	30 min
1.1.Cypermethrin	10 mg/kg	1	28.42	10:32	10:55	23 min
	50 mg/kg	1	30.98	10:33	10:45	12 min
	90 mg/kg	1	28.24	10:36	10:45	9 min

**Table 2:** Toxic severity (*s*) of tested chemicals computed at four hour after dosing

Test chemicals	Doses tested	Toxic severity (*s*) in %/sec
1.1.Dichlorvos	10 mg/kg	−199.0
	50 mg/kg	−19.8
	90 mg/kg	X
1.1.Chlorpyrifos	10 mg/kg	− 299.0
	50 mg/kg	−39.8
	90 mg/kg	11.1
1.1.Cypermethrin	10 mg/kg	−199.0
	50 mg/kg	20.0
	90 mg/kg	33.3

^X^ *Represents lab Balb c mouse which died earlier than the time for toxic severity evaluation*

**Table 3:** Toxic reaction rate (*r*) of tested chemicals computed at four hour after dosing

Test chemicals	Doses tested	Approximate length of time undesired effect significantly manifested	Toxic reaction rate (*r*) in mg/sec
1.1.Dichlorvos	10 mg/kg	60 min	−19.9
	50 mg/kg	30 min	−9.9
	90 mg/kg	15 min	X
1.1.Chlorpyrifos	10 mg/kg	2:30 h	−29.9
	50 mg/kg	1:30 h	− 19.9
	90 mg/kg	30 min	10.0
1.1.Cypermethrin	10 mg/kg	25 min	−19.9
	50 mg/kg	12 min	10.0
	90 mg/kg	9 min	30.0

^X^ *represents lab Balb c mouse which died earlier than the time for blood specimen collection*

**Table 4:** Changes in concentration of serum immunoglobulins at four hour after treatment of Balb-c mice with different doses of test chemicals

Test chemical	Tested doses	Quantitative immunoassay before treatment as reference test		Quantitative immunoassay at four hour after treatment for comparison		*ΔIg* serum conc.
		IgG	IgM	IgG	IgM	*Δ Ig*
1.1.Dichlorvos	10 mg/kg	< 1100 mg/L	70 mg/L	< 1100 mg/L	90 mg/L	+ 20 mg/L
	50 mg/kg	< 1100 mg/L	70 mg/L	< 1100 mg/L	80 mg/L	+ 10 mg/L
	90 mg/kg	X	X	X	X	X
1.1.Chlorpyrifos	10 mg/kg	< 1100 mg/L	90 mg/L	< 1100 mg/L	120 mg/L	+ 30 mg/L
	50 mg/kg	< 1100 mg/L	50 mg/L	< 1100 mg/L	70 mg/L	+ 20 mg/L
	90 mg/kg	< 1100 mg/L	90 mg/L	< 1100 mg/L	80 mg/L	− 10 mg/L
1.1.Cypermethrin	10 mg/kg	< 1100 mg/L	70 mg/L	< 1100 mg/L	90 mg/L	+ 20 mg/L
	50 mg/kg	< 1100 mg/L	80 mg/L	< 1100 mg/L	70 mg/L	− 10 mg/L
	90 mg/kg	< 1100 mg/L	80 mg/L	< 1100 mg/L	50 mg/L	− 30 mg/L

^X^represents treated mouse which died much earlier than the time for blood specimen collection

### Adverse effects of doses prepared from Dichlorvos, Chlorpyrifos and Cypermethrin

#### Dichlorvos pesticide

Three Balb c mice treated with different level of doses of Dichlorvos (10, 50 and 90 mg/kg body weight) were developed slow respiration and hypo-activity immediately after oral administration (Table 5). Lethal effect was observed at 1:36 h after oral treatment of Balb c mice with the dose at 90 mg/kg of test chemical. The signs and symptoms of adverse effect was manifested almost at the same time but with different magnitude for the three different level of doses. Slow respiration and hypo-activity was significantly manifested within 15 to 60 min after treatment depending on the amount of test chemical administered in the oral route. A sign of recovery such as increased activity with the survived two Balb c mice was observed about seven hours after treatment with 10 and 50 mg/kg of Dichlorvos. Even though the two Balb c mice look completely recovered in the 3rd day after treatment, they remained with significant loss of body weight on the 5th day after treatment (Table 6).

**Table 5:** The effect of different doses of Dichlorvos on Balb c mice which were administered orally using interagasteral tube

Dose in mg/kg	10	50	90
Number of mice treated	1	1	1
Adverse effects within 24 hrs	❖ Hypo-activity ❖ Weak ❖ Slow respiration	❖ Hypo-activity ❖ Weak ❖ Slow respiration	❖ Hypo-activity ❖ Weak ❖ Slow respiration ❖ Died after 1:36 hours
Adverse effects within 48 hrs	❖ Increased activity	❖ Slightly increased activity	
Adverse effects within 72 hrs	❖ Recovered	❖ Slightly recovered	
Adverse effects within 96 hrs	❖ Completely recovered	❖ Recovered	
Adverse effects within 120 hrs	❖ Weight loss	❖ Weight loss

**Table 6:** Body weight of Balb c mice which was weighed before and five days after treatment

Test chemicals	Doses tested	Weight before dosing	Weight on 5th day after dosing
1.1.Dichlorvos	10 mg/kg	15.13 g	13.37 g
	50 mg/kg	17.63 g	14.72 g
	90 mg/kg	16.42 g	X
1.1.Chlorpyrifos	10 mg/kg	30.41 g	26.58 g
	50 mg/kg	27.12 g	23.37 g
	90 mg/kg	26.84 g	X
1.1.Cypermethrin	10 mg/kg	28.42 g	23.58 g
	50 mg/kg	30.98 g	X
	90 mg/kg	28.24 g	X

^X^Represents laboratory Balb c mice which were died earlier than two days after dosing

#### Chlorpyrifos pesticide

Three Balb c mice treated with three different level of doses at 10, 50 and 90 mg/kg of Chlorpyrifos pesticide were also evaluated with acute toxicity trial for five days (Table 7). The main toxicity signs and symptoms significantly manifested after treatment were salivation, lacrimation, miosis (pinpoint eyes), trembling, breathing difficulty and general weakness that had been clearly manifested within about 30 min to 3 h after treatment depending on the amount of dose administered orally. Salivation, breathing difficulty and trembling had been worse on Balb c mice treated with the highest two doses (50 and 90 mg/kg body weight) especially about three hours after oral administration of test chemical. The Balb c mice treated with the highest dose (90 mg/kg body weight) died at 12:36 h after treatment and the other two Balb c mice treated with the lower two doses (10 and 50 mg/kg body weight) developed a sign of recovery such as increased activity and reduced trembling and salivation in the second and third day after treatment respectively (Table 7). Even though the two Balb c mice treated with the lower two doses looked completely recovered in the fifth day after dosing orally, they were still having significant loss of body weight (Table 6).

**Table 7:** The effect of different doses of chlorpyrifos on Balb c mice which were administered orally using interagasteral tube

Dose in mg/kg	10	50	90
Number of mice treated	1	1	1
Adverse effect within 24 hrs	❖ Trembling ❖ Breathing difficulty ❖ Tearing, Salivating ❖ Weak	❖ Trembling ❖ Tearing, Salivating ❖ Breathing difficulty ❖ Very weak	❖ Forceful trembling ❖ Tearing, salivating ❖ Breathing difficulty ❖ Died after 12:36 hrs
Adverse effect within 48 hrs	❖ Weak ❖ Slightly recovered	❖ Breathing difficulty ❖ Very weak ❖ Trembling	
Adverse effect within 72 hrs	❖ Recovered	❖ Slightly recovered	
Adverse effect within 96 hrs	❖ Completely recovered	❖ Recovered	
Adverse effect within 120 hrs	❖ Weight loss	❖ Weight loss	

#### Cypermethrin pesticide

Three Balb c mice treated with three different level of doses at 10, 50 and 90 mg/kg of Cypermethrin pesticide developed signs and symptoms of toxicity within about 9 to 23 min depending on the amount of dose administered orally (Table 1). The main signs and symptoms of toxicity significantly developed after treatment with the three different level of doses were stomach distension, tremor and restlessness, breathing difficulty, salivation and bulging eyes. Salivation, breathing difficulty, tremor and restlessness had been worse on Balb c mice treated with doses at 50 and 90 mg/kg of test chemical especially about two hours after dosing. The Balb c mice treated with the highest and second highest doses (90 and 50 mg/kg body weight) died at 11:36 and 26:00 h respectively after treatment (Table 8). The Balb c mouse treated with the lowest dose at10 mg/kg developed a sign of recovery such as increased activity, eating and drinking in the third day. It was however remained with mild breathing difficulty in the third day after treatment in the oral route. Even though it had shown a sign of complete recovery in the 5th day, the body weight was still less than the weight it had before treatment (Table 6).

**Table 8:** The effect of different doses of Cypermethrin on Balb c mice which were administered orally using interagasteral tube

Dose in mg/kg	10	50	90
Number of mice treated	1	1	1
Adverse effect within 24 hrs	❖ Distended stomach ❖ Bulging eyes ❖ Salivating ❖ Breathing difficulty ❖ Tremor	❖ Distended stomach ❖ Bulging eyes ❖ Salivating ❖ Breathing difficulty ❖ Tremor	❖ Distended stomach ❖ Bulging eyes ❖ Salivating ❖ Breathing difficulty ❖ Tremor & died after 11:36 hrs
Adverse effect within 48 hrs	❖ Slightly improved but with still breathing difficulty	❖ Died after 26:00 hrs	
Adverse effect within 72 hrs	❖ Breathing difficulty		
Adverse effect within 96 hrs	❖ Slightly recovered		
Adverse effect within 120 hrs	❖ Weight loss		

### Report of quantitative immunoassay

IgG (immunoglobulin G) and IgM (immunoglobulin M) quantification tests had been conducted to evaluate the immune response against test chemicals administered to Balb c mice in the oral route. Except Balb c mice treated with the highest dose at 90 mg/kg of Dichlorvos which was died at 1:36 h after dosing orally, about 1 ml of blood specimen had been collected from each Balb c mic using micro test tubes before treatment as reference test and at four hour after treatment for comparison (Table 4). Quantitative immunoassay showed that lethal doses prepared from Chlorpyrifos and Cypermethrin significantly suppressed serum concentration of IgM which was evaluated at four hour after dosing with test chemicals. However, specific quantitative result for serum concentration of IgG was unable to get from serum specimens which were collected at four hour after dosing orally. It was rather reported that each serum specimens sampled for quantification test had less than 1.1 g/L (< 1.1 g/L) of IgG (Table 4). The study has also showed that high concentration of serum immunoglobulin M had significantly suppressed the toxic effects (toxic severity and toxic reaction rate) of tested chemicals which was administered into Balb c mice in the oral route (Tables 2 and 3).

The negative value of computed toxic severity and toxic reaction rate in Tables 2 and 3 respectively represents the magnitude of toxic effect of administered dose of test chemical which was negligible in the biological system of treated Balb c mice. In other words, the administered dose of test chemical with a value of negative toxic reaction rate and toxic severity had negligible adverse effect at the organismal level. This, however, doesn’t mean that tested chemical was safe at the cellular level of treated Balb c mice. The toxic effect of test chemicals at the lower level of doses is limited at the cellular level which could not be significantly manifested at the organismal level. The toxic effect starts with biochemical change that leads to cellular change which eventually leads to physiological changes in the organ system which could be detected as signs and symptoms of adverse effect at the organismal level depending on the amount of dose administered to study organism. This implies that the role of a dose is only to determine the magnitude of adverse effect and length of time at which the adverse effect of tested chemical could be manifested in treated organism.

## Discussion

### The dose and its biological and clinical effects

The dose literally refers to the amount of a substance, medicine or drug in the field of nutrition, medicine and toxicology respectively to be utilized at a particular time for certain biological or pharmacological purposes [14–16]. The dosage refers to the rate of application of a dose [14, 16]. A single dose of Dichlorvos, Chlorpyrifos and Cypermethrin at different amounts (10, 50 and 90 mg/kg body weight) were administered to nine lab Balb c mice in the oral route and an integrated biological approach was employed in the study to evaluate the acute toxicological property of test chemicals for a maximum period of 5 days. The approach used mathematical formulations mentioned earlier to compute biological responses as toxic severity and toxic reaction rate which were used to analyse the cause of toxicity of each doses administered to study animals. The study proved that the adverse effect of tested chemical was as a result of its toxic reaction rate in the biological process of treated Balb c mice.

The different structure of the body of living things (humans, animals, microorganisms and plants) is the metabolic by-product of ingested dose of substances from the environment that involves different metabolic pathways in which one chemical is bio-transformed into another chemical with multiple bio-transformation mechanisms [17]. Metabolism is the means of life sustaining chemical transformation within the living organism. These biologically activated chemical transformation allow living things to grow, and reproduce, helps to maintain their structures and normal physiology in the environment. The metabolic system of an organism reveals the poisonous or nutritious or medicinal nature of test substance either at the cellular or organismal level depending on the amount of substance ingested. Thus, the manifestation of harmful effect of a substance within the biology of an organism determined by the nature of its chemical component rather than by the amount of substance ingested. The amount of a substance ingested by the living organism could however speed up the time at which biochemical and physio-pathological changes could be manifested in the biological system of an organism. Since the higher dose could manifest its adverse effect within a short period of time and the lower dose after a longer period of time in the treated organism, the lethal dose (LD_50_) and effective does (ED_50_) of test substance has no scientific ground to declare at a point of time that the lower dose is safe and the higher dose is unsafe for life. Even if the adverse effect of the lower dose is not significantly manifested at the organismal level within a short period of time, its adverse effect is significantly manifested at the cellular level which could cause impact on the life of an organism in the long run. In the previous study, for instance, the test extract at 500 mg/kg body weight killed treated Balb c mice in the 9th day and the highest dose (5000 mg/kg body weight) killed them in the 4th day after dosing orally [4]. In this study also 50 mg/kg of test chemical prepared from Cypermethrin pesticide killed treated Balb c mice in the 2nd day whereas 90 mg/kg body weight of tested chemical killed treated Balb c mice in the 1st day after treatment. The study showed that the dose determined the length of time at which significant adverse effect could be manifested in treated organism. The adverse effect of test substance is however determined by the nature of its toxic component which ultimately determines the toxic reaction rate in the biological process of an organism which in turn determines the extent of toxic severity of test substance. The toxic reaction rate (*r*) is the administered dose (*d*) over the length of time (*t*) at which signs and symptoms of adverse effect is manifested minus concentration of serum immunoglobulins change (*ΔIg)* as counter response to the toxic effect of test chemical on treated study animal [13]. It could be defined mathematically as $\left(r=\frac{d}{t}-\varDelta Ig\ \right)\  mg/\mathit{\sec}$[13]. In this study, *t* was considered four hours at which blood specimens for immunoassay were collected after dosing. The toxic reaction rate of test chemical was higher in the higher administered dose and the shorter the length of time at which adverse effect manifested in treated Balb c mice and Vic versa. The length of time in the investigation of acute toxicology was an essential factor to determine the toxicity of a test substance. The longer the period of time at which the undesired biological effect of test substance manifested on treated organism, the smaller the dose and its toxic reaction rate.

The toxic severity (*s*) of test chemical is the toxic reaction rate (*r*) over the administered dose (*d*) multiplied by one hundred which is expressed in percent per unit time [13]. It could also be defined mathematically as $\left(s=\frac{r}{d}\times 100\right)$ %/sec [13]. The higher the toxic severity of a test substance, the higher the toxic reaction rate and the smaller the administered dose which caused undesired effect in the biology of treated laboratory animal within a short period of time and Vic versa. The toxic severity and toxic reaction rate of 50 mg/kg prepared from Cypermethrin was, for instance, higher than the toxic severity and toxic reaction rate of the same dose prepared from Dichlorvos and Chlorpyrifos within a shorter period of time.

During this study where the toxicological effect of three test chemicals (Dichlorvos, Chlorpyrifos and Cypermethrin) were evaluated on Balb c mic at the age of 10 weeks, the toxic reaction rate and toxic severity of each tested chemicals were calculated and recorded in different tables as presented under subsection 3.2 in the result section. The value of toxic reaction rate (*r*) indicated safety limit of test chemicals whereas the value of toxic severity (*s*) predicted the length of time at which adverse effect of test materials could probably be manifested on treated organism. The laboratory Balb c mice treated with the amount of dose whose toxic reaction rate was less than zero survived from death whereas those sampled Balb c mice treated with the amount of dose that had toxic reaction rate more than zero died at different length of time after treatment depending on the toxic severity of tested chemicals.

It could be a scientific fact to declare that a test substance is safe when the value of toxic reaction rate (*r*) is less than or equal to zero. This means that the administered test substance is successfully neutralized and harmonised with the biological process of treated organism. The result of toxic severity (*s*) of each administered doses showed that treated Balb c mice with different dose had no equal opportunity to exist in life but equal fate for death at different lifespan depending on the amount of test chemical administered orally. This means that the higher the toxic severity of test substance the shorter the lifespan of treated organism and Vice versa. At the same time, the higher the toxic reaction rate of test substance in the biology of treated organism, the higher toxic severity of test material will be which shortens the lifespan of an organism.

A test substance said to be toxic not only when it has caused death but also pharmacological mechanism against the biological process of a living thing which could manifest its undesired effect in the long run. Death refers to a living thing that has lost complete bio-physiological interaction with its environment due to impaired systemic network which is probably caused either by an etiologic agent or a disaster. Death is usually happen when the impaired part outweighs the viable part of a systemic network in the diseased organism. It is a complete bio-physiological discontinuation of life from its environment where it has been rooted for years and decades. The amount of an etiologic agent therefore determines the magnitude of adverse effect which in turn determines the length of time at which death could probably happen to a living thing.

All the three different level of doses prepared from three different test chemicals mentioned earlier (10, 50 and 90 mg/kg body weight of lab Balb c mice) manifested the toxic effect with different magnitude at different length of time depending on the amount of dose administered in the oral route. If the higher dose kills treated organism, the lower dose which is referred to as effective dose (ED_50_), is most likely to have a higher risk of health problem in the long run. There is no scientific ground to categorise a single test material as safe dose (ED_50_) and lethal dose (LD_50_) at a point of time during the experiment, in other words, the lower dose could not be safe for life when the higher dose is lethal. It might not be manifested at the organismal level like does the higher dose but it could be significantly manifested at the cellular level which perhaps impact the health of treated organism in the long run. It is most likely to be a waste of time and resources to categorise a single test substance as effective dose (ED_50_) and lethal dose (LD_50_) and proceed to the next phase of preclinical trial with inadequately validated data.

### The immunoglobulins: an indicator of health conditions

The term immunoglobulin refers to any of a class of proteins present in the serum and cells of the immune system which function as an antibodies [18]. Immunoglobulins play an essential role in the body’s defence mechanism against antigens [18].

The immune system is made up of a network of cells, tissues and organs that work together to provide protection to the organism from environmental agents such as microbes or chemicals, thereby preserving the integrity of the organism. The specific immunity is further divided into humoral immunity, the one involved with antibody, and cellular immunity, which is orchestrated by T cells [18]. Through a series of steps which is called the immune response, the immune system attacks harmful organisms and substances that invade the biological system of the body and cause disease [19]. When antigens are detected in the body, several types of cells work together to recognize them and respond [19]. These cells trigger the B lymphocytes to produce antibodies which are specialized proteins that lock onto specific antigens [19].

The normal biological component of the immune system is therefore the overall indicator of the wellbeing of an organism whereas the abnormality in the immune system is the indicator of unhealthy conditions of an organism. What is going wrong in the biology of an organism is ultimately reflected as a quick counter response from the immune system that could be detected as abnormal temperature, supressed appetite, and abnormal immunoglobulin concentration in the plasma, physical and biological disintegration and many more to mention depending on the antigen detected by the immune system [19].

When the administered test substance is absorbed and interacted with the biological system of an organism, it is not only the subjective effect but also other multiple effects that could be triggered in the body which is ultimately detected by the immune system. The administered test substance becomes harmful to the life of treated organism when the biological systems of the body has failed to neutralize and harmonise the chemical component of test substance with the molecular counter part of an organism. Thus, an integrated biological analyses in the earliest stage of preclinical trial is crucial to make adequate assessment about the general safety of test material which may help to avoid progressive trail of harmful test chemicals.

## Conclusions

The study revealed that the dose had never determined the toxicity of tested chemicals but the magnitude of adverse effect and length of time at which adverse effect manifested in treated Balb c mice. The adverse effect of tested chemicals was rather determined by the toxic reaction rate in the biological process of treated Balb c mice. Adequate length of investigation time in acute toxicology was essential to determine the toxic nature of tested chemicals at different level of doses. Finally, the three different level of doses prepared from each test chemicals disproportionately suppressed IgM depending on the amount administered orally.

## Abbreviations

d: Administered dose
ED_50_: Median effective dose
IgG: Immunoglobulin G
IgM: Immunoglobulin M
LD_50_: Median lethal dose
r: Toxic reaction rate
s: Toxic severity
Sec: Second
t: The length of time at which adverse effect manifested on study subject
Δ Ig: Change in concentration of serum immunoglobulins at four hour after dosing

## Author’s information

The author received a diploma in Medical Entomology and Parasitology from Uganda allied Health and Management Sciences and Bachelor of Science in Biomedical Sciences, specialized in pharmacology from Makerere University in Uganda.
